# Why Is Eradicating Typhoid Fever So Challenging: Implications for Vaccine and Therapeutic Design

**DOI:** 10.3390/vaccines6030045

**Published:** 2018-07-24

**Authors:** Yi-An Yang, Alexander Chong, Jeongmin Song

**Affiliations:** Department of Microbiology and Immunology, Cornell University College of Veterinary Medicine, Ithaca, NY 14853, USA; yy394@cornell.edu (Y.-A.Y.); ac936@cornell.edu (A.C.)

**Keywords:** *Salmonella* Typhi, typhoidal Salmonellae, virulence, typhoid fever, vaccines, healthy carriers, chronic infections

## Abstract

*Salmonella enterica* serovar Typhi (*S.* Typhi) and *S.* Paratyphi, namely typhoidal Salmonellae, are the cause of (para) typhoid fever, which is a devastating systemic infectious disease in humans. In addition, the spread of multidrug-resistant (MDR) and extensively drug-resistant (XDR) *S.* Typhi in many low and middle-income countries poses a significant risk to human health. While currently available typhoid vaccines and therapeutics are efficacious, they have some limitations. One important limitation is the lack of controlling individuals who chronically carry *S.* Typhi. However, due to the strict host specificity of *S.* Typhi to humans, *S.* Typhi research is hampered. As a result, our understanding of *S.* Typhi pathogenesis is incomplete, thereby delaying the development and improvement of prevention and treatment strategies. Nonetheless, to better combat and contain *S.* Typhi, it is vital to develop a vaccine and therapy for controlling both acutely and chronically infected individuals. This review discusses how scientists are trying to combat typhoid fever, why it is so challenging to do so, which approaches show promise, and what we know about the pathogenesis of *S.* Typhi chronic infection.

## 1. Introduction

Over 2600 *Salmonella enterica* serovars have been identified and classified based on their surface antigens, Lipopolysaccharide (LPS) O antigen and flagellar H antigen [1]. *Salmonella enterica* serovars are split into two groups based on disease outcomes in humans: typhoidal Salmonella and nontyphoidal Salmonella (NTS) [2,3,4,5]. *Salmonella enterica* serovar Typhi (*S.* Typhi) and *S.* Paratyphi are typhoidal Salmonellae causing life-threatening systemic infectious diseases in humans, typhoid fever and paratyphoid fever, respectively. Paratyphoid fever, however, often shows milder symptoms than that of *S.* Typhi infection [6,7]. On the other hand, *S.* Enteritidis and *S.* Typhimurium are the most common NTS responsible for self-limiting gastroenteritis in healthy people. In addition, while typhoidal Salmonellae are human-restricted, NTS are broad host-range serovars infecting both humans and animals [8,9,10,11].

**Figure 1:**
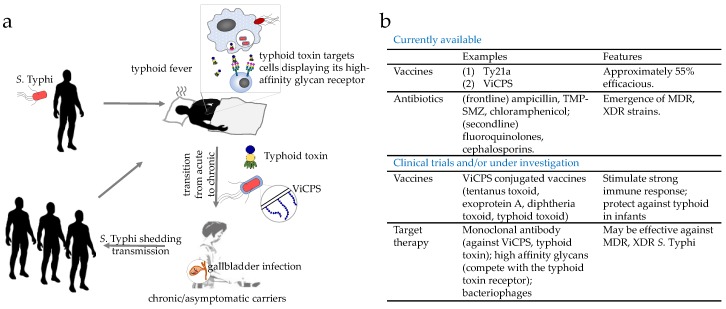
A cartoon depicting *S.* Typhi infection, disease development, transition from acute to chronic/persistent infection, and transmission. (**a**) Notable virulence factors (e.g., typhoid toxin and Vi capsular polysaccharide (ViCPS) of *S.* Typhi in disease development and transition from acute to chronic are discussed in Section 3. (**b**) A summary of prevention and therapeutic strategies against typhoid fever (details are discussed in Section 4).

*S.* Typhi is acquired through the ingestion of contaminated food and water, followed by invasion into the intestinal mucosa and its systemic spread to the liver, spleen, bone marrow, and in some cases gallbladder (Figure 1a). Symptoms and signs of typhoid include fever, headache, weight loss, lethargy, stupor, malaise, leukopenia, thrombocytopenia, gastrointestinal bleeding, and in some instances, neurological complications [12,13,14,15]. While some are characteristic of typhoid fever, most of these symptoms are also seen among patients suffering from other infectious diseases, indicating the need for appropriate methods to diagnose typhoid, such as culture, PCR and/or antibody-based detections of *S.* Typhi bacteria [16,17]. *S.* Typhi is estimated to affect at least 26.9 million people per year, of whom 1% die, even with appropriate antibiotic treatment [7]. Following recovery, the proportion of acute typhoid cases that become temporary carriers is estimated to be over 10%; further, a significant proportion of those individuals infected (2–6%) establish a chronic carriage state, during which they excrete *S.* Typhi for months and in a few cases for years [18,19,20] (Figure 1a). Prolonged or persistent infection of *S.* Typhi in macrophages and the gallbladder is known to be a key feature among these healthy chronic carriers [19,21] (Figure 1a). While lacking symptoms themselves, healthy carriers shed *S.* Typhi in their stool, which passes on the bacterium through the contamination of food and water sources. This is best exemplified by “Typhoid Mary,” or Mary Mallon, who caused a minimum of seven outbreaks of typhoid fever in her job as a cook during the 19th century (Figure 1a).

Therefore, to combat and contain *S.* Typhi, we would need to implement effective prevention and treatment strategies to control both acutely and chronically infected individuals. Two licensed vaccines that are commercially available are approximately 55% efficacious (Figure 1b). Although antibiotics are the primary treatment options for typhoid fevers, multidrug-resistant (MDR) and extensively drug-resistant (XDR) *S.* Typhi strains are spreading globally at an alarming rate (Figure 1b). As such, combating *S.* Typhi infections is a priority of the World Health Organization (WHO) and the Bill & Melinda Gates Foundation. This review discusses why eradicating typhoid fever is so challenging, advances in our understanding of the pathogenesis of chronic *S.* Typhi infections, and recent advances and emerging strategies in vaccine and therapeutic development against typhoid fever.

## 2. Why Is Eradicating Typhoid Fever So Challenging?

### 2.1. Epidemic of MDR and XDR S. Typhi

Since the 1970s, the emergence of MDR *S.* Typhi strains has continued to be the major barrier preventing successful control of typhoid fever in endemic areas [22,23,24]. The number of *S.* Typhi strains with resistance to ampicillin, trimethoprim-sulfamethoxazole (TMP-SMZ), and chloramphenicol has dramatically increased in the last two decades. For instance, from 1998 to 2010, there was an average of 14 typhoid fever diagnoses per year at a hospital in Malawi, of which 6.8% were MDR; in 2014, the number of typhoid patients increased to 782, and 97% of isolates were tested as MDR. Similarly, in Asia, the burden of MDR typhoid has been reported in many outbreaks, mostly in Pakistan, Vietnam, Nepal, and India [25,26,27,28]. Notably, the rapid spread of H58 *S*. Typhi haplotype has been linked to several MDR epidemics in sub-Saharan Africa and south/southeast Asia [29,30,31], which acquired *IncHI1* plasmids carrying antibiotic resistance genes. Due to the high prevalence of MDR *S*. Typhi, second-line antibiotics such as fluoroquinolones became the treatment of choice after conventional drugs were compromised. Sequentially, resistance to nalidixic acid and third-generation cephalosporins emerged, followed by chromosomal mutations in *gyrA*/*gnrS,* and acquisition of a plasmid that carries an extended-spectrum β-lactamase (ESBL) gene [31]*.* Recent epidemiological studies revealed a large proportion of H58 *S*. Typhi strains isolated in Pakistani patients since 2016 are XDR [31]. The XDR isolates were resistant to chloramphenicol, ampicillin, TMP-SMZ, fluoroquinolones, and third-generation cephalosporins, leaving very limited treatment options to these patients. Alarmingly, a recent study showed that XDR *S*. Typhi underwent rapid clonal expansion and sickened over 30 people endemically as well as international travelers [31]. It is undoubtedly an urgent need for scientists to develop better strategies to mitigate the possible XDR *S*. Typhi outbreaks in the future, including vaccines and novel treatment regimens.

### 2.2. Animal Models for Studying S. Typhi Pathogenesis

Both *S.* Typhi bacteria and some of its virulence factors (e.g., typhoid toxin) are adapted to humans. As a result, there is currently no optimal animal model that faithfully recapitulates most of the pathogenesis of *S.* Typhi infection. Despite these difficulties, several animal models, although each has its own limitations, allow us to study specific aspects of typhoid illnesses and *S.* Typhi infection.

#### 2.2.1. Animal Models for Acute Typhoid

**Figure 2:**
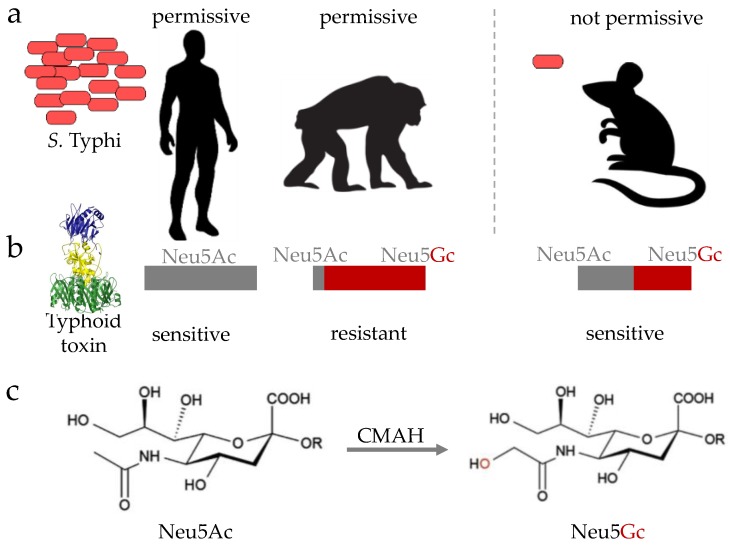
Cartoons depicting *S.* Typhi permissiveness of human and animal models (**a**), the expression of typhoid toxin’s glycan receptor (**b**). Chemical structures of Neu5Ac and Neu5Gc are shown in (**c**). cytidine monophosphate-*N*-acetylneuraminic acid hydroxylase (CMAH) converts Neu5Ac to Neu5Gc.

**Figure 3:**
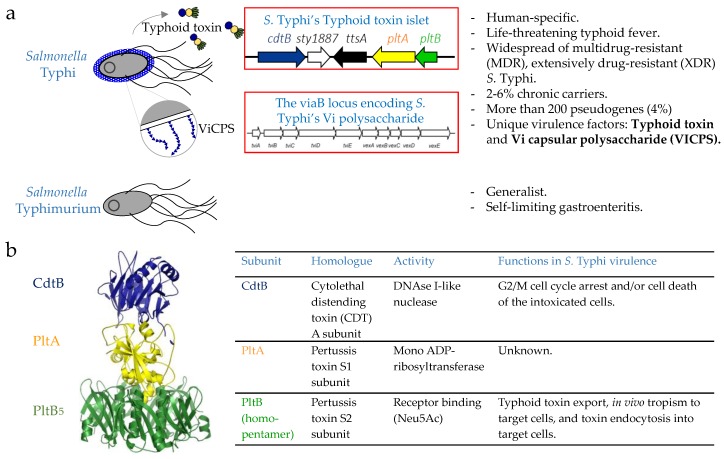
A summary of notable difference between typhoidal Salmonella and nontyphoidal Salmonella (NTS). (**a**) Different from *S.* Typhimurium, *S.* Typhi is human-specific and causes a life-threatening disease, typhoid fever. (**b**) Structure and function of typhoid toxin.

Besides humans, only higher primates such as chimpanzees are permissive of *S.* Typhi infection [32] (Figure 2a). Although chimpanzees experimentally support *S.* Typhi replication and share many genes with humans, *S.* Typhi-infected chimpanzees did not recapitulate typhoid symptoms, indicating that important host factors present in humans are missing in this animal model. One such host factor essential for the pathogenesis of *S.* Typhi is a glycan, notably *N*-acetylneuraminic acid (Neu5Ac) (Figure 2b,c). Neu5Ac is the host cell receptor for typhoid toxin, a distinct A_2_B_5_ toxin or exotoxin of *S.* Typhi that is secreted by intracellular *S.* Typhi during infection [33] (Figure 2b and Figure 3b). Unlike humans that exclusively express Neu5Ac, chimpanzees primarily express sialic acid *N*-glycolylneuraminic acid (Neu5Gc), which does not allow toxin binding to occur [34,35] (Figure 2b). This is because nonhuman animals have a functional enzyme termed cytidine monophosphate-*N*-acetylneuraminic acid hydroxylase (CMAH), which converts Neu5Ac to Neu5Gc (Figure 2c), thereby preventing toxin binding [34]. Consistent with observations that humans lack CMAH, while nonhuman animals contain it, humans are susceptible to typhoid fever disease, while other nonhuman primates, such as chimpanzees, are resistant to typhoid fever disease, despite their permissiveness for bacterial replication [32,35]. Mice do not, however, support *S.* Typhi replication, but naturally express the glycan receptor for typhoid toxin, Neu5Ac, despite the presence of CMAH. This is presumably due to its low expression in mice, indicating that mice administered with purified typhoid toxin serve as a surrogate model allowing for the study of the in vivo function of typhoid toxin that is thought to contribute to the acute symptomatology in typhoid patients [33,35,36]. In line with this notion, convalescent typhoid patients are shown to have high titers of anti-typhoid toxin antibodies in their sera [16,37,38]. Moreover, primary human immune cells express the specific glycan receptor for typhoid toxin [36]. CMAH knockout (KO) mice that exclusively express Neu5Ac, like humans, are also available [35,39], which is useful for pre-clinical testing of new, prospective preventative and therapeutic strategies against typhoid toxin-mediated symptoms and pathogenesis. 

NOD/SCID/γC^null^ (NSG) humanized mice infected with *S.* Typhi strain Ty2 have been shown to support rapid *S.* Typhi growth and cause death in infected mice, and therefore also serve as an acute typhoid model [40]. Another acute typhoid model is a mouse carrying a mutation of nramp1/slc11a1 that causes a typhoid fever-like illness and rapid death when infected by *S.* Typhimurium [41]. Nramp1/Slc11a1 is a divalent transition metal transporter (e.g., iron and manganese) involved in iron metabolism and host resistance to certain pathogens including *S.* Typhimurium [42]. In the case of *S.* Typhimurium infection, a homozygous mutant Nramp1^D169^ (refers as Nramp^-^ hereafter) is up to ~1000-fold more susceptible to bacterial infection [43,44]. This, in turn, allows us to understand the mechanisms that are shared between *S.* Typhimurium and *S.* Typhi. It is important, however, to note that we need to take caution when translating findings observed in *S.* Typhimurium to that of *S.* Typhi, as *S.* Typhimurium and *S.* Typhi are similar yet significantly different [2].

#### 2.2.2. Animal Models for Chronic/Persistent Typhoid

Several animal models have been established for studying chronic Salmonella infection. Nramp1^+/+^ (Nramp1^G169^) and Nramp1^+/−^ (Nramp^G169/D169^) mice are ~1000-fold more resistant to *S.* Typhimurium infection, compared to the Nramp1^−/−^ counterpart, and therefore serves as a chronic Salmonella infection model [45,46]. In this model with a lithogenic gallstone-inducing diet, Gunn and his colleagues demonstrated that gallstone biofilms facilitate gallbladder colonization and shedding of Salmonella [47]. Another chronic/persistent *S.* Typhi infection model is Rag2^−/−^γC^−/−^ humanized mice engrafted with human fetal liver hematopoietic stem and progenitor cells, where an important role of typhoid toxin in the transition of *S.* Typhi infection from acute to chronic has been identified [48]. The detailed underlying mechanism remains to be defined, but the in vivo binding preference of typhoid toxin—secreted by intracellular *S.* Typhi during infection—to immune cells suggests that an altered innate and adaptive immune response is likely responsible for this outcome [33,36].

#### 2.2.3. Human Infection Studies

Animal models are imperfect, which is particularly the case for human-restricted pathogens. To overcome the limitations that a model system presents, internationally concerted efforts are also centered on carrying out controlled human infection studies to advance our understanding of *S.* Typhi pathogenesis, to evaluate new conjugate vaccines, and to reveal useful biomarkers for acute or chronic typhoid [49,50,51,52]. Despite the many advantages, it is important to note that ethical concerns are a major limitation of human volunteer studies. More specifically, most human infection studies comply with stringent regulations, which limits infection doses and treatment/challenge period and intensity, thus limiting the scope of clinical studies [50]. In contrast, in the case of natural *S.* Typhi infections in endemic regions, patients often experience the full development of infection and disease and repeated infections, many of which would not be able to be investigated in human volunteers. Nonetheless, studies using various mouse and cell culture models, along with information obtained from human infection studies, have provided valuable insight into the pathogenic mechanism of *S.* Typhi, although much remains to be understood. Specifically, the mechanisms underlying chronic typhoid are one of the least understood research areas in *S.* Typhi infection.

### 2.3. Other Important Factors

To contain and eradicate *S.* Typhi infections, it is also important to improve and monitor water, sanitation, and hygiene (WASH) in endemic regions [53]. The importance of environmental factors in typhoid spread is evident from recent comprehensive analysis of the spatial and temporal distribution of typhoid infections in Dhaka Metropolitan Area of Bangladesh over the period 2005–2009 [54]. For instance, rainfall had a strong correlation with the occurrence of typhoid, with increasing *S.* Typhi transmission during the monsoon months [54]. Separately, temperature and river level were also correlated with an increase in typhoid incidence. Moreover, a statistically significant inverse correlation was found between typhoid incidence and distance to major waterbodies, but counterintuitively no difference between urban and rural environments. These indicate that water and sanitation upgrade would reduce the transmission of *S.* Typhi and emergence of new typhoid carriers, thus restricting the spread of disease. 

Moreover, a major obstacle in testing new, improved vaccines and therapies is a lack of known immunological correlates of protection in humans, which may be the consequence of issues discussed in Section 2.2, including a lack of animal models and ethical issues associated with human infection studies. For instance, simply analyzing the induction/alteration of certain immune responses may not be fruitful, as immunity generated by infection is not always protective (e.g., “cytokine storm” elicited by LPS), and in fact, relapses and reinfections can occur in individuals who show the elevation of certain immune responses [55,56]. Thus, further investigations are needed toward identifying immunological correlates of protection in humans, although new details have emerged about the complex adaptive host responses against *S.* Typhi in recent years [55]. Comprehensive analysis and discussion on current knowledge regarding the role of cell-mediated and humoral immunity following natural *S.* Typhi and *S.* Partyphi infections, experimental challenge, and immunization in humans can be found in a review paper by Sztein et al. [55].

## 3. Pathogenesis of Chronic *S.* Typhi Infection

### 3.1. Difference between Typhoid Salmonella and NTS

*S.* Typhi and *S.* Typhimurium share ~89% of their genomes [57,58]. *S.* Typhi has several virulence factors that are unique to *S.* Typhi and absent in NTS *S.* Typhimurium [57,58] (Figure 3a). Notably, *Salmonella* pathogenicity island 7 (SPI-7) contains the viaB locus that encodes genes involved in the synthesis and export of the Vi capsular polysaccharide (ViCPS) [2,59] (Figure 3a). One important function of ViCPS in *S.* Typhi virulence is inhibiting the Toll-like receptor 4 and 5-associated immune surveillance mechanism, as ViCPS hinders the surface exposure of lipopolysaccharide (TLR4 ligand) and flagellin (TLR5 ligand) in *S.* Typhi. As a result, the induction of host innate immune responses is prevented [59,60]. Another notable virulence factor of *S.* Typhi that is absent in *S.* Typhimurium is typhoid toxin [10,11] (Figure 3a,b). This toxin plays a multifaceted role that is pivotal in *S.* Typhi pathogenesis (see Section 3.2.3 typhoid toxin). Of note, several NTS strains, including *S.* Javiana, encode typhoid toxin homologs, which are 99% identical to its *S.* Typhi counterpart ([61] and manuscript in preparation). This 1% amino acid sequence variation is situated in the receptor binding PltB subunit and PltA subunit. This variation, in turn, results in no to little toxicity on cells expressing typhoid toxin’s high-affinity glycan receptors at the systemic site [36]. On the other hand, this sequence variation enables this toxin homolog to preferentially bind glycan receptors abundantly expressed on small intestinal epithelial cells (manuscript in preparation). These results are in line with the observations that NTS are restricted to the small intestine in healthy people, while typhoidal Salmonellae disseminate systemically.

Another notable feature of the *S*. Typhi genome is the presence of more than 200 pseudogenes (approximately 4% of its genes), which is thought to contribute to its narrow host specificity [57]. In *S.* Typhimurium, which is able to infect both humans and animals, approximately 0.9% of genes are pseudogenes [58]. Some of the pseudogenes found in *S.* Typhi are effectors that are secreted through the type III secretion system (T3SS). The T3SS is an essential virulence determinant in both *S.* Typhi and *S.* Typhimurium virulence, comprising the needle-like multiprotein apparatus, effectors, and chaperones [62]. The regulation and function of the T3SS Salmonella pathogenicity 1 (SPI-1) and SPI-2 are well characterized; they exert pivotal roles in invasion and survival/replication during *S.* Typhimurium infection, respectively [62,63,64,65,66,67,68,69,70,71]. Although the T3SS is common between *S.* Typhi and *S.* Typhimurium, significant variations between typhoidal Salmonella and NTS exist. For instance, unlike in *S.* Typhimurium infection, the importance of SPI-2 in *S.* Typhi pathogenesis is unclear, although a few SPI-2 effectors appear to be important during competitive growth in human macrophages [72,73]. Moreover, half of Salmonella T3SS effectors (of ~40 effectors) are pseudogenes or absent in *S.* Typhi [57,74]. In some cases, an effector(s) is expressed but loss-of-function by a mechanism different from pseudogenization. The smaller functional effector repertoire in *S.* Typhi is likely associated with its human-restricted lifestyle. For instance, GtgE is an effector with protease activity that is absent in *S.* Typhi, but present in *S.* Typhimurium. Intracellular *S.* Typhimurium in the Salmonella-containing vacuole (SCV) within mouse macrophages translocates GtgE into the host cell cytoplasm through a T3SS, where GtgE cleaves host cell Rab32 to create the cellular environment favorable for *S.* Typhimurium to grow in mouse macrophages [75]. Consistently, mice lacking Rab32 are permissive, although not fully, to *S.* Typhi infection [75]. The expression of *gtgE* in *S.* Typhi overcomes host cell restriction and enables colonization of mice, thus broadening host specificity. However, additional factors should contribute to host restriction, because the virulence of *S.* Typhi expressing GtgE did not match that of *S.* Typhimurium. Further, SptP, a SPI-1 effector possessing GTPase-activating protein (GAP) and tyrosine phosphatase domains, is a loss-of-function effector in *S.* Typhi. In *S.* Typhimurium, however, SptP plays an important role in mediating recovery of the host cytoskeleton post-infection [76]. Despite these functional differences, *S.* Typhi SptP is 94% identical to its *S.* Typhimurium counterpart. The 6% sequence variation is mostly situated within its chaperone SicP binding domain. As a result, its binding to SicP is hindered, causing SptP instability, preventing SptP translocation from bacteria into the host cytoplasm, and therefore restricting the intracellular activity of SptP [77]. In summary, the acquisition of new virulence factors and the smaller functional effector repertoire observed in *S.* Typhi appear to be consistent with its distinct lifestyle (i.e., systemic dissemination) and narrow host usage.

### 3.2. S. Typhi Pathogenesis with Emphasis on Chronic/Persistent Infection

#### 3.2.1. Importance of Understanding *S.* Typhi Persistence

Upon infection of *S*. Typhi, the host activates its innate and adaptive immune responses to clear the foreign invaders. However, many successful pathogens such as *S*. Typhi have evolved numerous strategies to survive the host immune response and persist for months and years. These chronic carriers, often asymptomatic, play a pivotal role in the continued disease transmission of this human-restricted pathogen, despite implemented public health interventions. Through the presence of undiagnosed carriers, *S*. Typhi continues to circulate in endemic areas. Therefore, scientists also direct their attention to searching for biomarkers and developing appropriate detection methods to identify chronic carriers [78,79,80,81]. Consistently, the WHO has identified this particular research area of *S.* Typhi infections as a priority to support further improvement in typhoid control [53].

The establishment of chronic/persistent *S*. Typhi infection is a complex process that involves multiple bacterial and host factors. Although the detailed in vivo molecular mechanisms have yet to be understood, both ViCPS and typhoid toxin play a vital role in this process. ViCPS, for instance, prevents the TLR4 and TLR5-associated host surveillance mechanism, while typhoid toxin targets immune cells, subsequently depleting or altering host innate and adaptive immune responses. This evidence suggests that they are import in organ/tissue penetration and the transmission of *S.* Typhi.

#### 3.2.2. ViCPS

ViCPS is expressed on the surface of virulent *S.* Typhi (Figure 3a). At the molecular level, genes responsible for the synthesis and transport of ViCPS to the bacterial cell surface are encoded by ten genes clustered and located on the viaB locus within the SPI-7 [59]. Among the ten genes, tviB, tviC, tviD, and tviE encode enzymes involved in the biosynthesis of Vi polysaccharide in sequential order, resulting in a linear polymer of α-1,4-linked *N*-acetylgalactosaminuronate [82]. While tviA is a transcriptional regulator for this operon, the other five genes, vexA, vexB, vexC, vexD, and vexE, are responsible for transport of ViCPS. The expression of ViCPS is regulated over the course of *S.* Typhi human infection, which appears to be correlated with the establishment of chronic/persistent infection of *S.* Typhi; clinical studies showed that *S.* Typhi chronic carriers contained high anti-Vi antibody titers in their sera [83,84]. Among the multiple roles that ViCPS has, one of the most crucial is assisting *S.* Typhi’s prolonged presence by concealing pathogen-associated molecular patterns (PAMPs), such as LPS and flagellin, from host immunosurveillance mechanisms [59]. ViCPS appears to effectively trick the host surveillance by multi-level regulations. For instance, TviA, a transcriptional regulator, not only controls the expression of genes involved in Vi capsular polysaccharide biosynthesis, but also inhibits flagellin translocation across the *S.* Typhi membrane [85], which appears to be favorable for *S.* Typhi in avoiding the TLR5-mediated host surveillance mechanism. Moreover, human clinical data support observations that ViCPS protects *S.* Typhi from being destroyed by host bactericidal activities [59,86,87]. Likewise, Vi-negative *S.* Typhi has been shown to be extremely human serum sensitive, as more than 99% of Vi-negative *S.* Typhi cells, for example, were rapidly lysed after exposure to human serum [88].

#### 3.2.3. Typhoid Toxin

Typhoid toxin plays a significant role during both acute and chronic infections [10,33,48,89]. This toxin displays a unique A_2_B_5_ stoichiometry, consisting of two enzymatic “A” subunits, CdtB (nuclease) and PltA (mono adenosine diphosphate (ADP)-ribosyltransferase), linked to a homopentamer of PltB (receptor-binding “B” subunit; Figure 3a,b) [33]. CdtB is a DNAse I-like nuclease that induces DNA damage and host cell cycle arrest and/or cell death [33,90,91]. If this intoxication process is not intercepted, cell death can result. PltA, on the other hand, is a mono ADP-ribosyltransferase for an unknown host target(s) [33,91]. The expression and membrane trafficking mechanisms of typhoid toxin are distinct. Typhoid toxin genes are expressed exclusively by intracellular *S.* Typhi located in the SCV within *S.* Typhi-infected host cells [91]. After expression, this toxin is encased in vesicles and trafficked out from the SCV to the extracellular environment; this process is triggered by the interaction of PltB with a Neu5Ac-bearing receptor(s) on the SCV membrane [92]. During this exocytic trafficking pathway, typhoid holotoxin does not have access to the host cell cytoplasm, indicating that the endocytosis of the secreted toxin to host cells is required for CdtB and PltA to carry out their functions [33,35,36,91].

The second-stage trafficking pathway, therefore, is the glycan (Neu5Ac) receptor-mediated endocytosis of the secreted holotoxin from outside cells into the host cell cytoplasm [33]. Typhoid toxin can endocytose both infected and uninfected neighboring host cells [91,92]. PltB is the receptor-binding subunit, which recognizes the specific trisaccharide consensus, N-acetylneuraminic acid (Neu5Ac)–galactose (Gal)–*N*-acetylglucosamine (GlcNAc) [33,35,36]. Although this consensus can be displayed by various types of glycoproteins and glycolipids on host cell membranes (both plasma and vesicle), PltB preferentially binds the trisaccharide consensus displayed by multiantennary N-linked glycoproteins (providing multiple Neu5Acs), as opposed to linear N-linked glycans displaying a single Neu5Ac, resulting in a high-affinity multivalent interaction between the PltB homopentamer and the glycan receptor [36]. Using this high-affinity binding, PltB plays an essential role in the exocytic and endocytic pathways of typhoid toxin [33,35,36,92]. In live mice, after administration of typhoid toxin to a systemic site to mimic its secretion during *S.* Typhi infection, the toxin targets immune cells and endothelial cells of arterioles in the brain, as they express the high-affinity multivalent N-linked glycan receptors for typhoid toxin [36]. Presumably, this in vivo tropism of typhoid toxin to immune cells (in particular, immune cells in the vicinity of *S.* Typhi-harboring macrophages, a primary reservoir of *S.* Typhi during infection) results in altered innate and adaptive immune responses, thereby creating an in vivo environment favorable for organ penetration and maintenance of *S.* Typhi, which then promotes chronic/persistent infection of *S.* Typhi. This result conforms to observations that innate and adaptive immune responses are essential for the host to control *S.* Typhi infection [93,94].

#### 3.2.4. Gallbladder Infection

Gallbladder infection of *S.* Typhi is a key feature of chronic typhoid [19]. *S.* Typhi forms bile-mediated biofilms on human gallstones [47]. Consistently, biofilm formation on gallstones during persistent *S.* Typhimurium infection in a Nramp1^+/+^ mouse fed a lithogenic diet resulted in enhanced fecal shedding and enhanced colonization of gallbladder tissue and bile. Unlike NTS *S.* Typhimurium, *S.* Typhi seems to exploit this rather harsh environment during persistent infection. In *S.* Typhi, T3SS SPI-1 associated genes were upregulated upon exposure to bile, resulting in a significant increase in epithelial cell invasion [95]. In *S.* Typhimurium, the opposite consequences, downregulation of SPI-1 genes and a repressed invasion into epithelial cells, were observed [95]. Unfortunately, at present, gallbladder removal is the only effective treatment for chronic typhoid carriage. Our understanding of chronic *S.* Typhi infection is incomplete, and much remains to be understood. Bridging this knowledge gap is vital to moving forward to the next level in combating *S.* Typhi infection.

## 4. Vaccines and Therapeutics against *S.* Typhi Infection

### 4.1. Typhoid Vaccines

#### 4.1.1. Typhoid Vaccines

Vaccination is one of the most efficient strategies to combat infectious diseases. Two typhoid fever vaccines are currently commercially available: the live oral vaccine Ty21a and the Vi polysaccharide vaccine (ViCPS or Vi) [96,97,98]. Ty21a is a mutant strain of *S*. Typhi Ty2 that is unable to survive in host cells due to mutations primarily in *galE* and ViCPS [97,98,99]*. galE* encodes for the enzyme uridine-diphosphate-galactose-4-epimerase (UDP-Gal-4-epimerase). In the absence of UDP-Gal-4-epimerase, galactose accumulates in the bacterial cell, ultimately making Ty21a an attenuated vaccine strain of *S*. Typhi [97,98,99]. Moreover, recent genome sequencing of the vaccine strain Ty21a revealed a total of 679 single nucleotide polymorphisms (SNPs) [100]. This vaccine is approved for use in individuals ages five and older, and it induces both cell-mediated and humoral immune responses against *S.* Typhi [96,97,98]. Moreover, the Ty21a vaccine-induced antibodies are able to cross-react against *S*. Paratyphi A and B, thereby indirectly indicating that the Ty21a vaccine may provide some protection against paratyphoid fever [6,97,101]. Nonetheless, efficient vaccines against *S.* Paratyphi are not currently available, although experimental paratyphoid vaccines are under investigation [97,101].

The ViCPS vaccine is based on the purified capsular polysaccharide, *S.* Typhi Vi antigen [96]. *S.* Typhi and *S.* Paratyphi C express ViCPS, but *S.* Paratyphi A and B do not. *S.* Paratyphi A is the primary cause of paratyphoid fever in endemic areas, which indirectly indicates that the potential cross-protection of ViCPS against paratyphoid fever in endemic areas is very limited. This vaccine induces a T-cell independent humoral immune response [96]. Its safe use for children as young as two years old underscores the importance of this vaccine, as children are at higher risk of *S.* Typhi infection in endemic areas [9,96]. Systemic review and meta-analysis of randomized controlled clinical trials revealed that the cumulative efficacy for the Ty21a vaccine is 51% and 55% for the ViCPS vaccine [102]. Based on the above, it is tempting to speculate that the primary reasons for this modest vaccine efficacy, among many, are likely associated with the poor ability of the Ty21a vaccine eliciting anti-Vi antibody titers, while most *S.* Typhi during natural infection expresses ViCPS on its surface. In the case of the ViCPS vaccine, it induces T-cell independent immune responses, thus resulting in a lack of prolonged protection. This can be improved by covalently conjugating ViCPS to carrier proteins, which enables the ViCPS conjugate vaccines to induce a T-cell dependent humoral immune response even in young children.

Accordingly, current international efforts on typhoid vaccine development are centered on designing more efficacious vaccines that can also protect children under age two. Moving towards this goal, several conjugate subunit vaccines combining ViCPS with another protein antigen (typically inactive forms of bacterial exotoxins) have been developed and are under active investigation. Among them, ViCPS conjugated with tetanus toxoid (Vi-TT or Typbar-TCV) appears to be the most promising and has already been approved for private use in India and Nepal, and has been recently prequalified by the WHO [53,103]. Tetanus toxoid is a recombinant inactive form of tetanus toxin produced by *Clostridium tetani* [104]. The Vi-TT vaccine was able to stimulate strong immune responses in children, even those who were younger than two years old [103]. The first field estimate comparing the (sero)efficacy of ViCPS and Vi-TT showed that the risk of serologically defined typhoid infection was lower in participants randomized to Vi-TT than those receiving ViCPS in individuals ages two and older [103]. Importantly, the Vi-TT vaccine likely protects against typhoid in infants aged 6–23 months old, because similar levels of protective antibodies in infants after Vi-TT vaccination were observed when compared to that of individuals ages 2–45 [103]. In addition, infants younger than six months old can be protected through maternal immunization. Other studies have recently tested the efficacy of Vi-rEPA, another promising ViCPS conjugate vaccine, where ViCPS combined with a recombinant Exoprotein A from *Pseudomonas aeruginosa* [105]. There is evidence that Vi-rEPA may have the potential to be effective in children younger than two years old; a study on Vietnamese children showed that Vi-rEPA was immunogenic in children aged two to five, with an effectiveness rate at 91.5% [106]. Moreover, Vi-DT is also a promising ViCPS conjugate vaccine that is under active investigation [104]. Vi-DT is made up of ViCPS conjugated to recombinant diphtheria toxoid, an inactive form of diphtheria toxin produced by *Corynebacterium diphtheriae* [104]. Also, Vi-CRM_197_ (CRM_197_ is a non-toxic, genetically-detoxified mutant of diphtheria toxin) has been developed and subjected to multinational clinical trials that took place in Philippines, Pakistan, and India, which has shown less promising results in certain populations, although further investigations are required for this conjugate vaccine [107]. This less promising result (less promising in one group while promising in another group) is likely associated with genetic, nutritional, or other environmental factors, which led to the different levels of immunogenicity and seroconversion rates after booster doses [108].

Lastly, given the evidence supporting the use and efficacy of conjugated vaccine candidates and the crucial pathogenic function that typhoid toxin plays during *S.* Typhi infection, it is conceivable that a ViCPS vaccine conjugated with a recombinant typhoid toxoid would provide similar efficacy in individuals of various ages including infants. Further, it is likely that the prospective Vi-typhoid toxoid provides additional protection against *S.* Typhi infection, compared to other conjugate vaccines, which includes reducing typhoid symptoms and inhibiting the establishment of chronic *S.* Typhi infection. Other anticipated benefits of the Vi-typhoid toxoid vaccine include potential cross-protection against paratyphoid, as both *S.* Typhi and *S.* Paratyphi A express typhoid toxin. 

#### 4.1.2. Progress on Paratyphoid Vaccine Development

*S.* Paratyphi A and B cause paratyphoid fever in humans, although *S.* Paratyphi A is the most common serovar [14]. *S.* Paratyphi infections are estimated to affect at least 5.4 million people globally, which has been increasing in recent years, particularly in Asia [14]. There is no licensed paratyphoid vaccine, although several promising paratyphoid vaccines are under investigations. Similar to typhoid vaccines, paratyphoid vaccines in development are based on either whole cell live-attenuated strains or subunit vaccines based on repeating units of the LPS O-antigen conjugated to different bacterial protein carriers. For instance, CVD1902 has been developed as a live-attenuated, oral vaccine candidate for *S.* Paratyphi A, which has two independently attenuating mutations in *guaBA* and *clpX* [109]*.* The CVD1902 vaccine candidate has been shown to be safe and immunogenic in preclinical phase I trials [109]. Three subunit vaccines have been developed; *S.* Paratyphi-specific O-antigen of LPS, O:2, is conjugated to tetanus toxoid (O:2-TT), diphtheria toxin (O:2-DT), or CRM_197_ (O:2-CRM_197_). The O:2-TT subunit vaccine was found, in phase 1 and phase 2 trials, to be safe and immunogenic after one dose, although a booster antibody response was not evident after a second dose [110]. Both O:2-DT and O:2-CRM197 were shown to be immunogenic, but clinical testing of these vaccine candidates has not yet commenced [111,112].

### 4.2. Limitations of Currently Available Typhoid Vaccines

Although the vaccines against typhoid have been shown to be effective, there are still some limitations. These vaccines do not provide 100% protection against *S.* Typhi infection. Moreover, they do not protect against paratyphoid, although some cross-protection is anticipated. Also, the current vaccines do not protect against virulence factors secreted by bacteria, such as typhoid toxin. This is because typhoid toxin is exclusively produced by intracellular *S.* Typhi, while the Ty21a vaccine strain is unable to produce antigens that are exclusively expressed by intracellular *S.* Typhi, due to its attenuation [36]. By considering the anticipated function of typhoid toxin during acute and chronic typhoid in people—supported by animal experiments and human blood and tissue testing—a conjugate vaccine combining ViCPS and typhoid toxoid is expected to result in an increased efficacy against (para) typhoid.

### 4.3. Typhoid Treatment Strategies Alternative to Antibiotics

In addition to improved vaccines, therapeutics alternative to antibiotics are urgently needed to overcome the escalating global spread of MDR/XDR *S.* Typhi. Ideally, effective treatment strategies would target both the bacteria and secreted virulence factors (e.g., typhoid toxin).

#### 4.3.1. Strategies Targeting the Bacteria

One approach is boosting host immune responses to clear the infection, whose limitations include side effects due to increased immune responses and a lack of specificity against pathogens. However, targeted immunotherapy such as pathogen-specific monoclonal antibody (mAb) therapeutics is able to overcome some of these limitations. MAbs provide high specificity for their targets, which results in a minimal cross-reactivity to the host tissues and a low chance of disturbance to other resident beneficial microbes [113]. Such microbial specificity prevents the selection for drug-resistant microbes among non-targeted microbes, and makes the mAb therapy superior to current broad-spectrum antibiotics. In fact, this therapeutic strategy, serum therapy or passive immunity, was used to treat several infectious diseases during the pre-antibiotic era, including diphtheria and streptococcal infection [114]. Nowadays, mAbs have predominantly been utilized in the field of neoplastic and inflammatory diseases. Despite the benefits, there is only one mAb (Palivizumab) licensed for use against an infectious disease [115], while many are in various stages of development. In typhoid, it is conceivable that anti-ViCPS antibody in conjunction with mAbs against other abundant yet specific outer membrane proteins of *S.* Typhi would serve as mAb therapeutics targeting *S.* Typhi. Similarly, in *S.* Typhimurium, monoclonal antibodies targeting the O-antigen of LPS and porins have been successfully demonstrated to partially protect the mice from *S. Typhimurium* infection [116,117,118]. Another promising approach is using a virus, namely bacteriophage, to fight against bacteria. For instance, Vi bacteriophages had been used to treat typhoid patients in Canada, which was effective for some typhoid cases [119]. Moreover, a recent study showed the potential of therapeutic bacteriophages that can lyse XDR *S.* Typhi isolated from the Democratic Republic of the Congo [120]. A major limitation of these strategies, however, is the potential for bacteria to evolve, and thus become ineffective.

#### 4.3.2. Strategies Mitigating the Action of Secreted Virulence Factors

Bacterial exotoxins play a crucial role in pathogenesis. In *S.* Typhi, based on animal experimental results and data from typhoid patients and human tissue samples, typhoid toxin is thought to play a vital role in symptomatology and chronic infection in humans. The action of typhoid toxin during infection could be prevented by multiple strategies, and similar strategies can be applied for targeting other secreted virulence factors based on their mechanism of action. For instance, therapeutic neutralizing monoclonal antibodies (mAbs) are a promising strategy with many advantages, as mAbs are highly specific to target toxins with little to no adverse effect on host cells and beneficial microbes. Shiga-toxin IIB (Stx2B), a bacterial AB toxin or exotoxin secreted by Enterohemorrhagic *E. coli* (EHEC) O157:H7, is responsible for organ damage in the hemorrhagic colitis and hemolytic uremic syndrome (HUS) during EHEC infection. Several mAbs against shiga toxin were shown to be effective in neutralizing the toxin during the early phase of the infection, with no detectable adverse events in healthy human volunteers [121,122]. In addition, mAb-mediated anti-toxin strategies are proven to be highly effective against other AB toxins, including botulinum toxin, ricin toxin, and anthrax toxin [123,124,125]. Among typhoid convalescent patients, typhoid toxin CdtB antibodies were abundantly detected in their sera [16,37,38]. Our group has recently demonstrated that mice immunized with typhoid toxoid elicited high titers of anti-CdtB antibodies that effectively protected mice against a lethal dose challenge of active typhoid toxin [36]. Based on the above, developing mAbs specifically targeting typhoid toxin seems to be a promising therapeutic for typhoid patients, especially when antibiotic-resistant strains are a rising concern.

Equally promising strategies against bacterial exotoxins include those that intervene with the interaction between toxins and their host receptors and/or their cellular trafficking mechanism (endocytosis or internalization). These would, in turn, result in little or no toxin delivery to the target place of their action. Most bacterial AB toxins (consisting of an enzymatic “A” subunit(s) and receptor binding “B” subunits), including typhoid toxin, use a specific type of glycan as their host cell receptor, suggesting that higher affinity glycans are able to compete over the endogenous host cell receptor displayed on host cell surface membrane in binding. Likewise, a higher affinity glycan appears to be a promising therapeutic in intervening with typhoid toxin that has a homopentamer receptor-binding B subunit PltB with total five binding pockets per toxin [33,35,36]. Similarly, in a study done on shiga-like toxins, a higher-affinity analog of their carbohydrate receptor—with five arms and two trisaccharide receptors attached to each—indeed effectively inhibited shiga-like toxin-mediated clinical symptoms [126]. Moreover, synthetic high-affinity glycoprotein glycans terminated with Neu5Ac have been shown to effectively compete over natural ligands, thereby inhibiting biological processes such as axon outgrowth [127]. Small molecule inhibitors that prevent retrograde toxin endocytosis processes could be used alone or in combination with glycan inhibitors, and would thereby inhibit host cells from being exposed to these toxins. These membrane trafficking modulators can also be combined with other modulators to redirect these toxins for degradation (e.g., mAb for extracellular toxins or another cell trafficking modulators to redirect the toxins to lysosomal or proteasome degradations). For instance, two small molecules in a chemical library screen were found as transport-inhibitor candidates that were able to block shiga toxin and cholera toxin transport [128,129]. These studies support the notion that small cell trafficking inhibitors could be useful in targeting typhoid toxin.

## 5. Conclusions

The global spread of MDR and XDR *S.* Typhi poses a great risk to human health. While the concerted international efforts on typhoid vaccine development significantly improve protection against typhoid fever, *S.* Typhi continues to spread and causes outbreaks in many areas. This is, in part, due to a lack of efficient strategies to take control of *S.* Typhi-carrying populations who are often asymptomatic and shed *S.* Typhi for months and years. Therefore, as an important initial step to contain and ultimately eradicate *S.* Typhi, we need to better understand the pathogenic mechanism by which *S.* Typhi establishes persistent and chronic infections, as it would offer insight into the rational design of improved vaccines and new therapies against *S.* Typhi infection.
